# Between Support and Risk: The Dual Role of Peer Relationships in Adolescents’ Mental Health

**DOI:** 10.3390/children12111569

**Published:** 2025-11-18

**Authors:** Maria João Carapeto, Inês Agostinho, Luísa Grácio, Daniela Santos

**Affiliations:** 1 Comprehensive Health Research Centre (CHRC), Universidade de Évora, 7004-516 Évora, Portugal; 2 Escola de Ciências Sociais, Universidade de Évora, 7004-516 Évora, Portugal; ines.agostinho@uevora.pt (I.A.); mlg@uevora.pt (L.G.); m60960@alunos.uevora.pt (D.S.); 3 Centro de Investigação em Educação e Psicologia (CIEP), Universidade de Évora, 7005-345 Évora, Portugal

**Keywords:** bullying, adolescence, peer group, well-being, social coping, help-seeking

## Abstract

**Highlights:**

**What are the main findings?**

- Youth perceived peers as both positive and negative influences on adolescents’ well-being and mental health.
- The perceived influence of peers was consistent with existing research on risk, promotive, and protective factors, but some adolescents may construe as positive certain peer relationships that serve as a means of avoidance or escape from problems.

**What is the implication of the main finding?**

- Understanding adolescents’ perceptions of peers as either positive or negative influences on mental health is essential to inform the development of peer-led programs aimed at preventing mental health problems and promoting well-being.
- Further research is needed to examine the adequacy of adolescents’ beliefs regarding the (mal)adaptive nature of various dimensions of peer relationships.

**Abstract:**

**Background/Objectives**: Adolescence is a developmental stage marked by profound transformations and heightened vulnerability to mental health difficulties, with peer relationships playing a central role, as they provide both protective and risk factors for adolescents’ mental health and well-being. This study aims to characterize Portuguese adolescents’ perceptions of how peers and friends influence their mental health, both positively and negatively. **Methods**: Participants were 99 adolescents aged 14–19 years old enrolled in a Portuguese secondary school. Data were collected through two open-ended questions and participants responses were analyzed using a mixed-methods approach, qualitative and quantitative. **Results**: Bullying and aggression were the most frequently reported negative influences, followed by peer pressure for inadequate behavior, toxic or false friendships, lack of support, criticism, and social exclusion. Conversely, social support was the most cited positive influence, alongside connection and belonging, promotion of emotional well-being, positive peer characteristics, and social learning. **Conclusions**: Findings support the dual role of peer relationships, which may either exacerbate vulnerability or strengthen resilience. The study underscores the importance of school-based prevention strategies that reduce bullying and peer aggression while fostering prosocial climates and supportive peer interactions.

## 1. Introduction

Adolescents’ mental health has received increasing attention in scientific literature and public health policies, reflecting the concern with the recent epidemiological data and its importance for integral human development. Adolescence, the period between 10 and 24 years of age [1], corresponds to a stage of profound physical, emotional, cognitive, and social transformations. This developmental period is characterized by identity construction, the establishment of meaningful interpersonal relationships, and the progressive assumption of increased responsibilities [2]. It is, therefore, a transitional stage which, by combining multiple changes, entails both a high developmental potential and heightened vulnerability [3,4]. During this stage, the emergence of mental health problems such as anxiety, depression, and suicidal ideation is frequent, with a direct impact on adolescents’ well-being and overall functioning [5]. A deeper understanding of risk and protective factors, as well as of adolescents’ perceptions regarding their difficulties and help-seeking, is essential for effective prevention programs and mental health promotion strategies in this population.

At the international level, it is estimated that over 13% of adolescents live with mental disorders, with anxiety and depression being the most prevalent–often comorbid–and especially among girls [5]. Depression, in particular, constitutes a central risk factor for suicide, being the third leading cause of death among young people [5]. Around 75% of mental health disorders emerge before the age of 24, highlighting adolescence as a critical stage for early intervention and prevention [6]. Despite the high prevalence of mental health problems, only a minority of adolescents seek professional help [7].

Adolescent mental health is influenced by a set of risks, promotive and protective factors within nested individual, family, and social systems [8]. These factors operate interdependently, either amplifying psychological vulnerability or, conversely, fostering resilience and well-being, and are therefore fundamental determinants of the developmental process and of healthy adjustment during this stage of the life course [3,9,10]. These multiple, interacting systems underscore that resilience is not a static trait but a dynamic process of positive adaptation within changing contexts [10].

Research highlights the role of certain risk factors in the development of adolescent mental health problems (e.g., anxiety, depression, suicide and self-harm, deviant behavior). At the individual level, risk factors include pre-existing mental health issues, loneliness, poor sleep, restrictive dieting, addictive behaviors, low self-esteem, conflicts in self-knowledge, perfectionism, body dissatisfaction, insecure attachment, and difficulties in emotion regulation, among others [4,9,11,12,13,14,15,16,17]. Family-related risks have also been identified, such as low socioeconomic status, caregivers’ mental health issues, poor relationships with caregiver, family conflict, adverse childhood experiences, and dysfunctional parenting [11,15]. Contextual factors such as negative school climate and high academic burden seem also to increase adolescents’ vulnerability [11,15]. Although less studied, some protective factors have been pointed out such as healthy sleep and diet, physical activity, emotional regulation, strong family support, and positive family functioning [11,15].

Peer relationships assume a central role during adolescence, acting as both risk and protective factors for adaptive development, well-being and mental health. Viewed through an ecological lens, the impact of these relationships is embedded within a broader network of interconnected contexts, including family, school, community, and cultural settings, which collectively shape adolescents’ experiences, identity formation, social behaviors, and mental health [18]. Peer relationships are crucial for providing opportunities for social interaction, cooperation, and the development of social skills [19], and fulfill fundamental needs for acceptance, inclusion, self-reliance, and credibility in adolescence [20]. However, these interactions are not uniformly positive, as they can also involve peer pressure, contribute to negative peer affiliations, or result in victimization and antisocial behaviors [20].

While peer victimization and bullying [21], chronic rejection and exclusion [22], peer pressure to engage in risky behaviors (e.g., substance use, self-harm, delinquency; [18,23]), co-rumination and emotional contagion [24], contagion of suicidal ideation or self-harm [15], and social comparison [25], among others, may threaten adolescents mental health and well-being, peers also contribute to adolescents’ better adaptive outcomes. Examples are the protective effects offered by good quality of emotional support [26], sense of belongingness [27], buffering against stress and adverse events [17], peer modeling of positive behavior [28], friendship [29], peer acceptance [30], identity formation through peers [31], or peer encouragement of help-seeking [32]. Moreover, the quality of peer relationships appears to influence how adolescents cope with developmental challenges. For example, relationships characterized by support and trust tend to foster more adaptive coping strategies, whereas interactions marked by conflict, rejection, or interpersonal stress increase the likelihood of relying to avoidance strategies [33]. In addition, avoidant coping has been found to be strongly associated with perceived threats to one’s skills, autonomy, and one’s own sense of belonging, particularly in situations involving peer-generated stress [34]. Thus, peer relationships may shape adolescents’ coping with stressful events in both adaptive and maladaptive ways.

However, only a limited number of studies offer insights into adolescents’ own perspectives on the positive and negative influences of peers on their mental health. According to the adolescents’ perceptions, they recognize as protective factors trusting relationships with peers and friends [35,36]. Family support is perceived as a primary protective factor, but peers are the most immediate source of support and help seeking targets, especially for mild mental health problems [35]. Adolescents tend to value peers and friendships characterized by understanding, loyalty, attentiveness to emotions, and non-judgmental listening, with trust-based bonds contributing to the mitigation of feelings of isolation and to the strengthening of perceived social support (Johns Hopkins Bloomberg School of Public Health & United Nations Children’s Fund [37]). Peers who assist in solving current problems and provide encouragement in times of doubt are also regarded as important contributors to mental health during difficult times [37].

From adolescents’ perspectives, a range of additional risk factors for mental health have been frequently reported. Alongside bullying and peer aggression, they emphasize the negative influence of peer pressure, particularly in relation to substance use, risky behaviors, and social exclusion, which can exacerbate vulnerability to emotional distress [37]. Moreover, adolescents view lack of trust and fear of negative judgment from peers as barriers to self-disclosure, which hinder their willingness to seek help [7].

This knowledge about the risk and protective factors perceived by the adolescents within peer relationships is crucial for the development of effective, youth-centered mental health promotion, prevention, and treatment strategies. Adolescents are not merely passive recipients of psychosocial influences; they actively interpret, negotiate, and respond to their environments, in particular in this developmental stage when they develop their autonomy and identity separately from their caregivers and invest more trust in their peers. Their subjective perspectives offer valuable insights that can complement epidemiological data and theoretical models in the development of effective preventive programs [38,39]. In addition, both the literature and adolescents’ perceptions underscore trust and open communication as crucial in help-seeking, with adolescents valuing informal (especially peer) networks, while also recognizing the relevance of formal, professional support [37].

An integrated understanding of risk and protective factors within the context of peer relationships, including adolescents’ views, enable the design of prevention strategies that enhance protective resources, such as trust-based bonds and prosociality, and that promote help-seeking [7,40,41,42]. This is the case of Stronger Youth—“Empowering young people social competences and soft skills through peer mentoring” [43], an Erasmus+ project aiming to develop a peer mentoring program to be implemented in school settings, promoted by a consortium of seven organizations from six European countries (Check Republic, Italy, Poland, Portugal, Romania and Spain). The current study was developed specifically within the context of this project, with the objective of incorporating the perspectives of adolescents from different countries into the decision-making processes underlying the design of the peer mentoring program.

In addition, adolescents’ perceptions of peers’ positive and negative influences on well-being and mental health can be understood within the framework of the World Health Organization’s Global Action for Measurement of Adolescent Health [44] indicators, which emphasize both health outcomes and their social determinants. Negative peer influences may increase the risk for mental health difficulties, such as GAMA indicators on depression, anxiety, and emotional distress. On the other hand, supportive peer relationships can foster resilience and subjective well-being, reflecting indicators on social connectedness and perceived social support. Moreover, adolescents’ views provide insight into proximal determinants of coping behaviors and adaptive strategies, helping to explain the pathways through which peer interactions impact mental health. Therefore, considering adolescents’ lived experiences of peer influence is crucial for understanding mental health development and for guiding interventions and monitoring in line with global frameworks.

Thus, the present study had the following main research goals: (1) To describe the negative influences (or risk factors) for adolescents’ mental health that Portuguese adolescents perceive within the context of peer and friendship relationships; (2) To characterize the positive influences (protective and promotion factors) for adolescents’ mental health that Portuguese adolescents perceive within the context of peer and friendship relationships.

## 2. Materials and Methods

### 2.1. Participants

The study sample comprised 99 adolescents aged between 14 and 19 years (*M* = 15.67, *SD* = 1.23), all enrolled in a secondary school located in southern Portugal. Of these, 53 were female (53.54%), 44 male (44.44%), and two identified as “other” gender (2.02%). With respect to school grade, 33 participants (33.3%) were in the 9th grade (final year of basic education), 35 (35.4%) in the 10th grade, 12 (12.1%) in the 11th grade, and 18 (18.2%) in the 12th grade (one missing response). Among secondary school students, the majority were enrolled in the Science and Technology program (*N* = 45; 45.5%), followed by the Languages and Humanities program (*N* = 14; 14.1%). A smaller number attended the Economics program (*N* = 6; 6.1%), and one participant did not provide information.

### 2.2. Instrument

Data were collected through a broader questionnaire, designed within the framework of the Stronger Youth project about adolescents’ perspectives on adolescents’ mental health difficulties and resources, and preferred help-seeking channels. It also contained a series of open-ended questions addressing the perceived influence of key life contexts in adolescents’ lives, including peers’ context, on adolescents’ well-being. Open-ended questions were used to capture adolescents’ personal perspectives in their own words. Although written responses to open-ended items may vary in length and depth, and some participants may provide limited detail, this approach allows for the collection of richer, more nuanced data than closed-ended formats. It offers adolescents the opportunity to express their experiences and meanings more freely, providing valuable contextual insights [45,46]. To minimize social desirability bias in responses, participants’ anonymity and confidentiality were ensured. It was explicitly stated that there were no right or wrong answers, and the questions were formulated to refer both to adolescents’ own experiences and to those of others. In addition, a self-administered questionnaire format was employed [47]. In particular, this study focused on two open-ended questions addressing the perceived positive and negative influences of peers for adolescents’ mental health (i.e., the perceived promotive and protective, and the risk factors, respectively): “Considering both your personal experience and that of other adolescents you know, how does the peers and friends influence the well- being of adolescents? Give examples of how peers and friends can contribute positively to the well-being of adolescents”, and “Now, give examples of how the peers and friends can contribute negatively to the well-being of adolescents”.

### 2.3. Procedures

The study employed a primarily qualitative approach, combining content analysis with descriptive statistics of the resulting categories. This approach was preferred because it is well-suited for capturing adolescents’ personal perspectives in their own words, as well as potential contextual subtleties.

A convenience sampling strategy was applied in a secondary school in southern Portugal, which agreed to collaborate in the Stronger Youth project, including this data collection. The sampling process focused on adolescents in grades 9 through 12, because this developmental stage has been shown to involve heightened vulnerability to mental health issues (such as depression and anxiety; [48]). Furthermore, adolescents in this developmental range are more capable of reflecting meaningfully on the risk and protective factors they perceive [49]. The study was presented to class directors of 9th to 12th grade classes, and eight of twenty teachers arranged for their students to participate outside regular class time. A total of 101 adolescents volunteered and completed the questionnaire. Two participants were excluded because they did not answer the questions relevant to this study.

Prior to data collection, authorization was obtained from the school principal. An informed consent form describing the objectives, methodology, and guarantee of anonymity was distributed to students for parental or legal guardian signature and subsequently returned to the research team. Paper-and-pencil questionnaires were administered in classrooms, out of regular class time, with the assistance of school psychologist and class director, both of whom received brief training to support the administration of the questionnaires and data collection process.

The study complied with national and international ethical guidelines for research involving human participants and received formal approval from the Ethics Committee of the University.

### 2.4. Data Analysis

Qualitative responses were transcribed into digital files. A categorical system was developed to structure the content analysis of adolescents’ responses [50]. Two independent coders initially reviewed all the responses for each question and proposed a set of four to seven content categories, for question. In a subsequent meeting with the coders and two additional researchers, these proposals were discussed until a consensual coding framework was established. Six categories were defined from the responses to the question about positive influences, and seven categories emerged from the responses to the question about negative influences, including an “other” category to capture material outside the predefined codes.

The same two coders then applied this system of categories to analyze the content of the participants’ responses. Responses to the questions on positive and negative influences were each examined for content corresponding to the respective categories. Following the independent coding phase, intercoder reliability was computed using Holsti’s method [51], which is equivalent to the percentage agreement measure when both coders code the same items. The resulting reliability coefficient was 0.92 for the positive influences of peers and 0.97 for the negative influences, indicating a high level of agreement between coders. In addition, the coders met with the researchers to reconcile discrepancies until consensus. The number of participants mentioning contents of each category was counted, and the respective percentages were then calculated.

Table 1 and Table 2 present the final set of categories along with their coding criteria for each of the two questions.

## 3. Results

Table 3 presents the risk factors for well-being identified by the adolescents.

The category Bullying and Aggression is the most frequently reported as risk factor for adolescents’ mental health, addressed through themes such as bullying or other forms of aggression, as ridicule (e.g., “They may make fun of the situation”, P. 11), disdain, disrespect, and all actions with the intention of humiliating or harming others (e.g., “‘Friends’ who mistreat others”, P. 14). This is followed by Pressure for inappropriate behavior, expressed through reactions that primarily have behavioral effects, such as encouraging risk behaviors, poor decision-making, exerting control, and manipulation of others (e.g., “They may influence us to the point where we no longer make decisions ourselves, which are not of our own choice”, P. 23). The category Toxic, False Friendships encompasses relationships that have a detrimental effect on one’s emotional state (e.g., “Sometimes the people (peers and friends) around us may be toxic, which can affect our well-being”, P. 41), as well as references to false friendships, relationships based on self-interest, and betrayal (e.g., “Envy, intrigues”, P. 35). Lack of Support is expressed through insufficient behaviors from others when one is in need of support (e.g., “Ignoring the signs that a peer is giving, not paying attention when a peer opens up”, P. 16), such as lack of assistance, denial of help, failure to listen, and lack of empathy, which may result in loss of trust and feelings of insecurity. Criticism, Judgment, and Comparisons not only includes these themes but also encompasses some peers’ cognitive distortions perceived as unfair misinterpretations or misconceptions or abusive conclusions (e.g., “Judging a book by its cover”, P. 58). The category Other comprises responses such as “I don’t know” or answers that could not be categorized elsewhere (e.g., “The opposite of what I mentioned above”, referring to the other question, P. 72). Finally, Social Exclusion and Isolation refers to themes such as distancing, exclusion, leaving others aside, or lack of integration (e.g., “putting people aside, when at times they just do not want to be alone”, P. 38).

Table 4 presents the factors identified as protective and promoting adolescents’ well-being.

The category Social Support is addressed through themes such as helping, providing support, offering good advice, listening to problems, giving guidance, confiding, and problem-solving (e.g., “Listening to our concerns; helping us to resolve, or attempting to resolve, our problems”, P. 3), among others. Connection and Belonging involves spending time together, maintaining contact, interaction, companionship, socializing (e.g., “Providing company, acting as a family without being family.”, P. 13), inclusion, identification, and conversation. Promotion of Emotional (Hedonic) Well-being is expressed through themes referring to improvements in emotional state resulting from peer interaction, such as positive affect, feelings and emotions, distraction from problems, enjoyment, and stress relief (e.g., “When they help us to relieve some of the stress that school brings.”, P. 29). Characteristics of Peers involve attributes that foster an environment conducive to another’s well-being, such as loyalty, friendship, honesty, and enabling others to feel safe, understood, or able to be vulnerable (e.g., “Someone who understands and supports them.”, P. 38). Social Learning refers to learning from peers, offering positive role models, and interpersonal development (e.g., “Influences the practice of engaging in regular physical exercise.”, P. 80). The category Other encompasses responses, about uncertainty or lack of knowledge, or those that do not fit within the remaining categories (e.g., “Our friends are themselves; they are not the ones who solve our problems. It depends on our own ability to make friends; either we toughen up, or we are in trouble—pardon the expression.”, P. 12).

## 4. Discussion

This study aimed to describe the perspective of Portuguese adolescents about the influence, both positive and negative, of peer contexts on adolescents’ well-being and mental health. Main results highlight adolescents’ ability to identify both positive and negative influences of peer relationships in adolescent mental health, reinforcing the hypothesis of their ambivalent role of peer relationships also reported in other research. In general, Portuguese adolescents’ views on peers positive and negative influences on adolescents’ mental health and well-being were consistent with those of adolescents from other countries [35,37] and with research on risk and promotive, protective factors (e.g., [9,11]). Bullying and aggression were the risk factors most frequently reported by adolescents, whereas social support emerged as the most salient promotive, protective factor.

The most consensual perceived positive influence was peer support when an adolescent faces psychological challenges, which is in line with studies evidencing the positive role of friendship and quality of support on adolescents’ well-being and mental health [17,26,29]. Similarly, the literature robustly emphasizes the protective role of supportive relationships, which facilitate emotional regulation, resilience, and help-seeking behaviors [40]. The prominence of social support goes along with the peers’ characteristics that adolescents particularly value, like trust, loyalty, and understanding. This aligns with the findings of [35], who describes peers as the most immediate source of support for young people, often preceding family members or healthcare professionals. Consistently, the lack of support was perceived as a negative influence.

Bullying and aggression was the risk factor perceived by most adolescents. These findings are consistent with previous studies demonstrating that exposure to school bullying is associated with a higher likelihood of mental health problems, including anxiety, depression, and suicidal ideation [21,52]. Issues as toxic or false friendships, criticism, and comparison also emerged, reflecting adolescents’ heightened vulnerability to social evaluation, an aspect also highlighted in the literature on psychosocial and brain development [4,53]. Conversely, the power of interaction with peers, especially friends, to promote emotional, hedonic well-being, and temporarily alleviate the stress or avoid and escape from problems was also mentioned, in accordance with other studies [54,55]. Coping in adolescence often involves seeking support, co-regulating emotions, and using social interactions to manage stress or adversity [56,57]. It appears that, beyond providing emotional comfort, peers also play a central role in shaping adolescents’ social coping strategies. For instance, Zimmer-Gembeck and Skinner [55] found that adolescents often rely on avoidance strategies to distance themselves from negative thoughts, perceiving this as a positive coping mechanism because it provides immediate relief. However, recent evidence indicates that although such strategies may temporarily reduce emotional tension, their habitual use is associated with reduced coping flexibility and greater emotional difficulties in the medium term. Furthermore, when adolescents perceive a threat in peer contexts, they tend to employ less effective coping strategies, leading to ineffective emotional regulation, whereas peers with greater flexibility are more likely to use adaptive strategies [55]. In the same direction, recent studies further highlight that adolescents increasingly engage in online activities, many times involving virtual interactions with peers, such as social media or video games, as a form of escapism from stress or negative emotions. Similarly, while these activities may provide short-term relief, peer-mediated online interactions can also reinforce maladaptive coping and risk behaviors (such as excessive screen time, cyberbullying, or exposure to harmful content) potentially exacerbating mental health difficulties [58,59]. This topic warrants further investigation, as adolescents may misconstrue the perceived benefits of avoidance or escape from problems, coping strategies that research has linked to increased mental health difficulties (e.g., [55]).

The second most mentioned positive influence of peers was connection to peers and sense of belonging, highlighting the protective role of integration within social groups. This finding is in line with the Unified Theory of Development [60], which emphasizes the importance of family, school, and community contexts in the development of self-regulatory competencies [61]. It is also consistent with research demonstrating the mental health benefits of belongingness. For instance, the sense of belonging showed a buffering effect against the negative impact of low peer acceptance on loneliness and depression [27]. On the other hand, adolescents also mentioned the negative influence of social exclusion and isolation, in accordance with other studies (e.g., [22,27]).

An important topic that emerged both as positive and negative influence is related to social learning (e.g., [62]). Some adolescents perceived the beneficial effects of learning from peers as models of adaptive behavior and skills, and of interactions with peers as opportunities for personal development. Even more adolescents perceived the risk of peer pressure to perform maladaptive, risky behavior (tobacco or alcohol consumption, skip classes, etc.), reported by research as an actual possibility (e.g., [18]).

Overall, the findings support the hypothesis of ambivalence of peer relationships, previously evidenced by studies showing that they can function both as a risk [9] and as promotive, protective factor [63]. The present findings highlight the dual role of peers in shaping adolescents’ well-being and coping strategies. These findings can be understood in light of diverse theoretical perspectives, which help illuminate the mechanisms through which peer relationships contribute to adolescents’ resilience and vulnerability. From the perspective of social learning theory [62], as the adolescent participants noted, peers serve as models whose behaviors and coping strategies are observed and potentially imitated, which may explain why both adaptive and maladaptive patterns emerge in peer interactions. Attachment theory further suggests that high-quality peer relationships can provide additional sources of emotional security, supporting resilience in the face of developmental stressors [64]. These patterns can also be framed within developmental psychopathology and risk-protection balance models, which emphasize that peers constitute both potential risk factors (e.g., peer-generated stress or cyber-related harms) and protective factors (e.g., social support, guidance), shaping trajectories of adaptation [10].

A final topic deserving attention concerns the potential cultural specificities in adolescents’ perceptions of peer relationships. Portuguese sociocultural norms, which emphasize close social bonds and empathy, may shape how adolescents perceive peer interactions [65]. Supportive interactions are highly valued, whereas negative behaviors, such as exclusion, can have a particularly strong impact, highlighting a dual role of peers in influencing adolescents’ mental health. Moreover, different experiences of peer relationships in different countries [65], highlight the importance of considering sociocultural context when designing peer-led interventions.

### Study Limitations and Implications for Future Research and Intervention

Certain limitations of this study must be acknowledged. The sample, restricted to a specific school context and to adolescents who volunteered to complete the questionnaire outside regular class time, does not permit generalization of the results to other contexts, settings, or populations, therefore limiting the external validity of the conclusions. Data collection via self-report opens the possibility of biases, such as social desirability or omission of more negative experiences. Open-ended questions were used to capture adolescents’ perspectives in their own words; however, this format may introduce certain biases, despite the procedures undertaken to minimize them, as described in the Section 2.3. Responses may be influenced by adolescents’ verbal expression skills, motivation, and willingness to disclose personal views, as well as by social desirability concerns. Furthermore, variability in the length and depth of responses may result in unequal representation of participants’ perspectives. The questionnaire format does not allow for in-depth exploration of adolescents’ responses, as the interview format would.

Future research may benefit from larger and more diverse samples, as well as mixed method approaches that integrate quantitative and qualitative data. Finally, it would be pertinent to explore gender and age differences, as the literature suggests distinct trajectories in how boys and girls from different age groups experience and manage peer influence [5]. Among others, the issue of co-rumination deserves deeper inquiry. Repetitive discussions about problems among peers (particularly friends), without seeking solutions and with a strong focus on negative emotions or situations, have been identified as a risk factor for internalizing symptoms in adolescence [24] of which adolescents may be unaware.

These results have direct implications for the prevention and intervention on adolescent mental health. Firstly, they highlight the need for school-based programs that combine the reduction in bullying behaviors with the promotion of pro-social climates, in order to foster trust and supportive relationships among peers [40,66]. Secondly, they suggest the importance of empowering adolescents to distinguish healthy from toxic relationships, thereby strengthening their emotional self-regulation and decision-making skills [67]. Finally, peer-led interventions show promising effects on adolescents’ mental health and well-being [68]. Key elements of effective implementation include appropriate peer recruitment and training, ongoing supervision, and integration within school systems [69]. To optimize their impact, it is crucial to incorporate adolescents’ own perceptions of peer-related risk and protective factors, thereby ensuring that peer-led programs are grounded in the social realities that shape their coping and well-being. The findings about adolescents’ perceptions of peers’ positive and negative influences on mental health suggest a few recommendations for peer-mentoring programs [70], such as the Stronger Youth project. For example, the mentor selection process should consider both empathy and assertiveness, the latter allowing mentors to guide, support, and maintain appropriate boundaries with their mentees. Training for adolescent mentors and supervising educators should cover mental health literacy and an understanding of how peers can impact adolescents’ well-being and adaptive coping, both positively and negatively. Additionally, peer mentors should be trained to balance structured, developmentally appropriate activities with opportunities for open, informal communication within the mentor–mentee relationship.

Overall, investing in youth mental health fosters individual well-being and the development of more sustainable societies, with positive impacts on education, employability, and civic participation [71].

## Figures and Tables

**Table 1:** Categories and coding criteria of positive influences of adolescents’ mental health provided by the peers’ context.

Positive Influences	
Social support	Events in the interaction that include actions of listening, helping, supporting, encouraging and concerning and/or that allows to vent, talk, solve problems.
Promotion of emotional (hedonic) well-being	References to improvements in emotional state through interaction with peers that raise positive feelings/affections/emotions or momentary decrease or eliminate negative emotional states
Connection and belonging	Interactions that include spending time together and promote coexistence and feelings of belonging.
Social learning	General references to learning from others or more specific through good examples, learning to respect interpersonal development.
Characteristics of peers	That create an environment conducive to each other’s well-being allowing the other to feel safe sharing problems, feeling understood, or being vulnerable.
Others	Seeking a professional, saying they don’t know

**Table 2:** Categories and coding criteria of negative influences of adolescents’ mental health provided by the peers’ context.

Negative Influences	
Bullying and aggression	Bullying or other forms of aggression, mocking, contempt, or disrespect; mistreating, blackmailing, assaulting–anything intended to humiliate or hurt.
Pressure to inadequate behaviors	Other behaviors that have an effect primarily on a behavioral level leading to risky behavior “bad behavior”, poor decisions, and school performance Control over others’ lives, manipulation
Toxic and false friendships	Relationships that have a negative effect on one’s own emotional state Reference to false friendships, out of self-interest, Betrayal trust.
Lack of support	Insufficient support, help, empathy of the other (not disagreements). Effects of this behaviors on the individual
Social exclusion and isolation	Behaviors of isolation, exclusion, left aside.
Criticism, judgments and comparisons	Behaviors and attitudes of devaluation, negative comparison, criticism and distortion
Others	“I don’t know”, “the opposite of what I said previously”

**Table 3:** Perceived risk factors for adolescents’ well-being.

Categories	Example	*N*	%
Bullying and aggression	“Bullying, disrespect (…)” (P. 8)	45	44.45
Pressure for inappropriate behavior	“Influencing them to consume alcoholic beverages and to smoke” (P. 7)	32	32.32
Toxic, false friendships	“There are many peers who, when one confides in them, disclose our problems to others, and at that point trust is lost” (P. 2)	22	22
Lack of support	“Not helping us, and always wanting to be in a better position than us” (P. 19)	20	20.20
Criticism, judgment, and comparisons	“(…) criticizing our problems (…)” (P. 3)	9	9.09
Other	“No response.” P. 81; “If they are true friends, they will not contribute negatively to your well-being.” (P. 50)	5	5.05
Social exclusion and isolation	“Excluding from groups.” (P. 4)	5	5.05

Note. *N* = 99. P—participant.

**Table 4:** Promoting and protective factors for well-being.

Categories	Example	*N*	%
Social support	“Helping when needed (…)”, P. 1	63	63.01
Connection and belonging	“(…) spending time with us (…)”, P. 21	35	35.35
Promotion of emotional (hedonic) well-being	“(…) distracting our mind so that we are not constantly thinking about problems.”, P. 3	23	23.23
Characteristics of peers	“(…) loyalty; friendship; trust”, P. 9	23	23.23
Social Learning	“By setting good examples”, P. 93	6	6.06
Other	“I don’t know”, P. 25	5	5.05

Note. *N* = 99. P—participant.

## Data Availability

The data presented in this study are available on request from the corresponding author. The data are not publicly available due to the lack of participant permission for this specific use.
